# Altered PPP2R2A and Cyclin D1 expression defines a subgroup of aggressive luminal-like breast cancer

**DOI:** 10.1186/s12885-015-1266-1

**Published:** 2015-04-15

**Authors:** Francisco Beca, Miguel Pereira, Jorge F Cameselle-Teijeiro, Diana Martins, Fernando Schmitt

**Affiliations:** 1 IPATIMUP – Institute of Molecular Pathology and Immunology of the University of Porto, Rua Dr, Roberto Frias, s/n, 4200-465 Porto, Porto, Portugal; 2 Department of Medical Oncology, Dana-Farber Cancer Institute, 450 Brookline Ave., Boston, MA 02215-5450 USA; 3 Division of Surgical Oncology, Department of Surgery, Massachusetts General Hospital Cancer Center, 55 Fruit St, Boston, MA 02114 USA; 4 Complexo Hospitalar Universitario de Vigo (CHUVI), Rua de Pizarro, 22, 362004 Vigo, Spain; 5 IBMC – Instituto de Biologia Molecular e Celular da Universidade do Porto, Rua do Campo Alegre, 823, 4150-180 Porto, Portugal; 6 Department of Pathology and Medicine, Laboratorie National de Sante 1, Rue Louis Reche, L-3555 Dudelange, Luxembourg

**Keywords:** Breast cancer, Immunohistochemistry, TCGA PPP2R2A (B55α), Cyclin D1, Prognosis

## Abstract

**Background:**

PPP2R2A deletions were recently linked to a subgroup of luminal breast carcinoma (BC) that exhibits poor survival. This subgroup also exhibited amplification of a chromosome region containing the Cyclin D1 coding gene, CCND1. Therefore, we aimed to investigate whether a combination of PPP2R2A (B55α) and Cyclin D1 expression statuses evaluated by immunohistochemistry (IHC) could define a subgroup of luminal BC that exhibits poor survival.

**Methods:**

First we conducted a retrospective cohort study using sequencing data from The Cancer Genome Atlas initiative to correlate PPP2R2A copy number alteration (CNA) status with its expression level and the corresponding overall survival (OS). Next, also using a retrospective cohort study design, we evaluated the PPP2R2A (B55α) expression levels by IHC in a total of 807 BC patients from two independent cohorts (discovery cohort n = 349 and validation cohort n = 458). Cyclin D1 expression was also evaluated, and the PPP2R2A (B55α)^-/low^/Cyclin D1^high^ phenotype was evaluated as a predictor of disease-free survival (DFS) and OS in luminal-like BC patients.

**Results:**

Deletions in the PPP2R2A gene strongly correlate with lower mRNA expression and poorer OS. PPP2R2A (B55α)^-/low^ carcinomas have significantly shorter DFS and OS. Furthermore, in univariate analysis, the PPP2R2A (B55α)^-/low^/Cyclin D1^high^ phenotype is significantly associated with poorer DFS and OS. In a multivariate analysis, the PPP2R2A (B55α)^-/low^/Cyclin D1^high^ phenotype is significantly associated with poor DFS, thus defining a group of luminal-like BC with higher risk of relapse.

**Conclusion:**

We demonstrate that BCs harboring PPP2R2A deletions are associated with worse OS. Moreover, this is the first study to demonstrate that the combination of altered PPP2R2A (B55α) and high Cyclin D1 expression by IHC defines a subgroup of luminal-like BC patients with a high risk of relapse and death.

**Electronic supplementary material:**

The online version of this article (doi:10.1186/s12885-015-1266-1) contains supplementary material, which is available to authorized users.

## Background

PP2A (Protein Phosphatase 2) is a major heterotrimeric serine/threonine phosphatase that consists of one structural (PP2A/A), one catalytic (PP2A/C) and one regulatory (PP2A/B) subunit [1]. The PP2A/A and PP2A/C subunits each have two known isoforms (α, β) and comprise the core dimer (reviewed in [2]). Conversely, the PP2A/B subunit has multiple isoforms, and it is the major determinant of substrate specificity [3]. As a Ser/Thr phosphatase, PP2A counteracts the actions of Ser/Thr kinases, which are often defective or deregulated in cancer. Examples include components of the MAP kinase and AKT pathways, the tumor suppressors pRB and p53, and CDK1 substrates (reviewed in [2]).

Substantial evidence points to a role for PP2A as a tumor suppressor. Early studies showed that toxin-mediated inhibition of PP2A was a potent tumor-promoter stimulus and that PP2A had a pivotal role in modulating the transforming ability of oncogenic viruses [4,5]. More recent reports have demonstrated that PP2A is inhibited in colorectal carcinoma [6]. Such inhibition is due, at least partially, to the downregulation of some of PP2A regulatory subunits encoded by the PPP2R5E and PPP2R2A genes [6]. Alterations in PPP2R5E have also recently been shown to be a driver of clonal evolution in breast carcinoma [7]. Specifically, PPP2R2A is located on chromosome 8p21.2 and encodes B55α, a B regulatory subunit of PP2A. Loss of PPP2R2A is a common event in non-small cell lung cancer, and it results in the impairment of the homologous recombination repair pathway [8]. Additionally, shRNA-mediated silencing of PPP2R2A has been shown to be a potent oncogenic signal in experimental models of colon carcinoma [8]. Furthermore, in patients with acute myeloid leukemia (AML), low levels of B55α expression in blast cells have been associated with shorter complete remission duration, demonstrating that in AML, the decreased expression of PPP2R2A (B55α) is an adverse prognostic factor [9]. Taken together, these findings lead to the conclusion that PP2A, as well as some of its regulatory subunits, such as PPP2R2A, have definitive roles as tumor suppressors in a large variety of cancers.

In breast carcinoma (BC), the expression status and genomic alterations of PP2A and it subunits, such as PPP2R2A, are only now being elucidated. Despite recent reports of the possible therapeutic targeting of PP2A with inhibitors in BC [10], knowledge is still limited. Different haplotypes of PPP2R2A modify BC risk [11], but mutations in PPP2R2A are rarely detected in BC (The Cancer Genome Atlas Research Network BRCA dataset, provisional). More interestingly, in a recent study, copy number aberrations (CNAs) centered on PPP2R2A, along with heterozygous and homozygous deletions were commonly found in BC, and they were associated with a mitotically active estrogen receptor (ER)-positive BC subgroup [12]. The same study also identified an ER-positive BC subgroup, comprising 11q13/14 cis-acting luminal tumors that exhibited a steep mortality trajectory with elevated hazard ratios (Integrative Custer 2) [12]. The CNAs within this subgroup also contain the amplification of a region at 11q13 that harbors the Cyclin D1-encoding gene CCND1 [12]. Nevertheless, rapid translation of these findings to a clinical setting has been limited because of the need to use genomic and transcriptomic profiling, technologies that are not routinely available in most pathology laboratories.

Here, we test the hypothesis that PPP2R2A protein expression, detected using immunohistochemistry (IHC), will uncover a group of ER-positive BCs that exhibit a differential outcome. Additionally, we hypothesized that the combined assessment of PPP2R2A and Cyclin D1 protein expression would further refine the subgroups’ outcome associations and be potentially useful in identifying Integrative Cluster 2-like BC.

## Methods

### The Cancer Genome Atlas (TCGA) data

Data from the Invasive Breast Cancer dataset (TCGA, Provisional) of the TCGA database on PPP2R2A mRNA expression levels as determined by RNA sequencing (RNA Seq V2 RSEM - Illumina HiSeq 2000 platform), copy number alteration (CNA) as determined by GISTIC 2.0 analysis, recurrence status and overall survival (OS) were downloaded from the cBIO Cancer Genomics Portal on March 24, 2014 [13,14]. Survival analysis was performed by comparing patients with no CNAs with those with heterozygous or homozygous deletions.

### Patient samples

**Figure 1:**
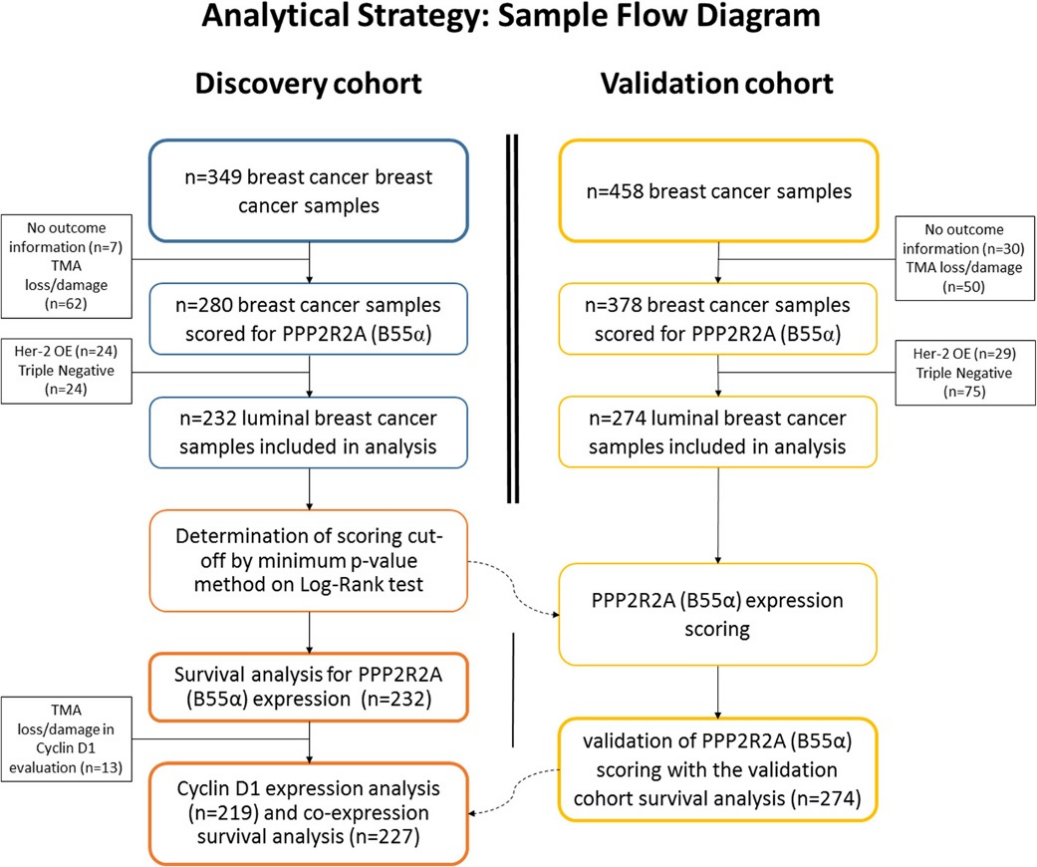
Diagram illustrating the analysis strategy and number of patients included at each step.

Additionally we used the two following independent cohorts of clinical samples: (a) a discovery cohort comprised of 349 non-consecutive, randomly selected patients with BC who had undergone surgery at Centro Hospitalar de São João, EPE, Porto, Portugal, between January 2001 and March 2006; (b) a validation cohort comprised of 458 non-consecutive, randomly selected patients with BC who had undergone surgery between 1978 and 1992 at the Hospital Xeral-Cíes, Vigo, Spain. Only patients with histologically confirmed BC from which adequate formalin-fixed, paraffin-embedded tissue was available were included. The tumor grade was determined according to the modified Bloom and Richardson classification [15]. The exclusion criteria were similar for both cohorts and included the following requirements: previous history of BC, hereditary BC, bilateral BC at diagnosis, refusal of surgical treatment, synchronous tumors and cases with neoadjuvant therapy as the initial treatment. All estrogen receptor (ER)-negative and lymph node (LN)-positive patients in the discovery cohort had received adjuvant chemotherapy. None of the HER2+ patients had received trastuzumab as the initial treatment. The clinical definitions used for OS and Disease-Free Survival (DFS) are detailed in the Additional file 1. Women in whom the envisaged end-point was not reached were censored as of the last follow-up date. Subjects with missing values for survival and PPP2R2A (B55α) expression in both cohorts were excluded from the analyses. A diagram of the complete analytical strategy and the flow of patients through the study, including the numbers of patients included in each stage of the analysis, are shown in Figure 1.

This study was conducted according to the Portuguese National Regulative Law for the handling of biological specimens from tumor banks (waiving the need of specify approval for samples from a tumor archive and used in retrospective analysis) and complied with The Helsinki Declaration statement.

### Tissue Microarrays (TMAs) and IHC

Representative areas of invasive BCs were selected on hematoxylin and eosin (H&E)-stained sections, and two tissue cores (2 mm in diameter) were obtained from each specimen for TMA construction as previously described [16]. An H&E-stained section from each TMA block was reviewed to confirm the presence of morphologically representative areas of the original lesions. Appropriate controls with normal breast tissue were also included. After pretreatment and antigen retrieval, the slides were incubated with the primary antibodies for PPP2R (sc-81606; Santa Cruz Biotechnology, diluted 1:50) and Cyclin D1 (SP4; Neomarkers, diluted 1:50). For the discovery cohort, clinicopathological data were obtained from original pathology reports and Ki-67 IHC performed in cases missing this information. ER, PgR, Her2 and Ki-67 IHC were performed on the validation cohort, and used as surrogates for molecular subtyping, based on 13th St Gallen International Breast Cancer Conference (2013) Expert Panel recommendations [17]. The cut-off level for ER and PgR receptor positivity was nuclear staining in more than 1% of tumor cells, and the cut-off level for Ki-67 was based on work described in Cheang *et al*. [18]. In both cohorts, HER2 evaluation was carried out in compliance with the most recent American Society of Clinical Oncology/College of American Pathologists (ASCO/CAP) guidelines [19]. The details regarding TMA construction, antibodies used and the detailed conditions for each antibody, including those targeting PPP2R2A (B55α) and Cyclin D1, are included in the supplementary data (Additional file 1: Table S1). All morphological and IHC assessments were made by a pathologist (FS). Cyclin D1 expression was determined as previously described [20].

### PPP2R2A (B55α) scoring

**Figure 2:**
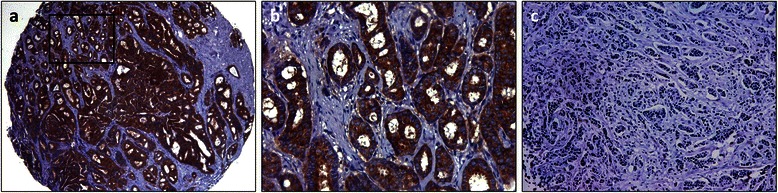
Two examples of PPP2R2A (B55α) IHC expression. PPP2R2A (B55α)^high^ expression in a low-grade carcinoma (**a**, 4×; **b**, 400×); PPP2R2A (B55α)^-/low^ expression in a high grade carcinoma (**c**, 100×).

Because to the best of our knowledge there is no published data regarding a PPP2R2A (B55α) scoring method in BC, we first used the discovery cohort to establish the ideal assessment criteria for PPP2R2A (B55α) expression by IHC and applied the same methodology to the validation cohort. Briefly, PPP2R2A (B55α) IHC expression was semi-quantitatively evaluated in the cytoplasm of invasive carcinoma according to previous methodology [21]. Afterwards, using the minimum *p*-value method on the log-rank test for OS in the discovery cohort, we determined the ideal cut-off for dichotomization of PPP2R2A (B55α) scoring (further details are provided in the Additional file 1) [22]. After establishing the IHC assessment criteria in the discovery cohort, expression in the validation cohort was determined using the same methodology. Briefly, tumors were considered PPP2R2A (B55α)^-/low^ when there was no detectable expression or if it was weak in less than 5% of tumor cells (Figure 2).

### Statistical analysis

Where appropriate, Pearson’s χ2, Fisher’s exact and Student’s t tests were used. Cumulative survival probabilities were estimated using the Kaplan–Meier method, and differences between survival rates were tested for significance using the log-rank test. Multivariate analysis for survival was performed using the Cox proportional hazards model. Variables included in the models were defined *a priori* and are indicated under each table. Departures from the proportional hazards assumption were assessed based on the Schoenfeld residuals. Hazard ratios (HR) and 95% confidence intervals (95% CI) were estimated for each variable. All tests were two-sided with a 95% CI and a *p*-value of < 0.05 considered significant. Correction for multiple comparisons was performed where indicated using the Benjamini-Hochberg procedure. TCGA data were analyzed using R Version 3.0.2 for Mac OS X, and all other statistical analysis were carried out using Stata® software, version 13.

All analyses and data reporting were performed according to the “REporting recommendations for tumour MARKer prognostic studies” (REMARK) guidelines and STrengthening the Reporting of OBservational studies in Epidemiology (Additional file 2) statement [23,24].

## Results

### PPP2R2A CNA status is associated with mRNA expression levels and OS in the TCGA dataset

**Figure 3:**
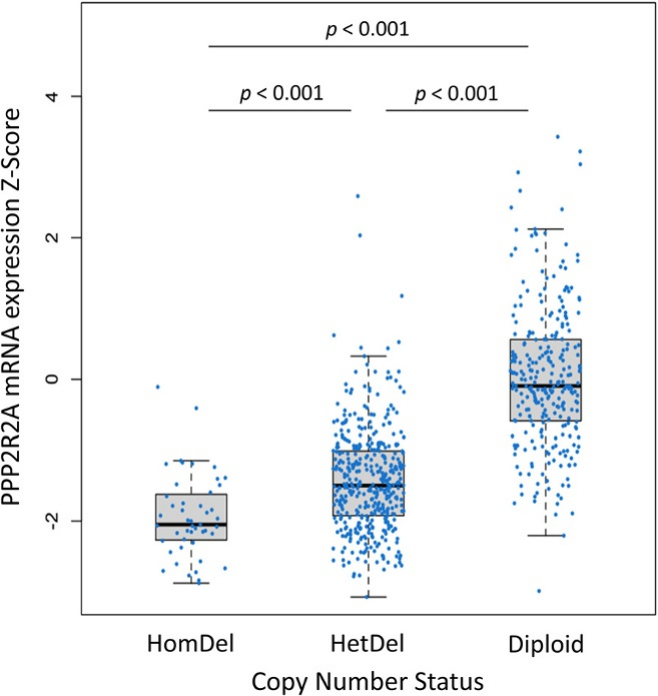
Expression levels of PPP2R2A according to CNA. Using data from the TCGA database, the expression levels of PPP2R2A were plotted according to the following classifications of gene copy number: normal (n = 278), heterozygous deletion (HetDel) (n = 411) or homozygous deletion (HomDel) (n = 47). Significant differential expression was observed for all pair-wise comparisons using Student’s*t*-test (*p*-value <0.001 for all comparisons), showing a positive relationship between copy number and gene expression.

To investigate whether there was a correlation between CNA and PPP2R2A expression, namely if CNA with any deletion was associated with lower PPPR2A expression levels, data for 852 BC samples were retrieved from TCGA. We selected the cases displaying either normal copy number, heterozygous PPP2R2A deletion or homozygous deletion (n = 736) for further analysis. Cases displaying either heterozygous or homozygous deletions at the PPP2R2A gene locus constituted 53.8% (n = 458) of all cases. The PPP2R2A mRNA expression level shows a significant association with CNA, with cases with heterozygous or homozygous deletion of the PPP2R2A locus displaying a significantly lower mRNA expression Z-Score (Figure 3). The *p*-values of the t-test performed for every pairwise comparison were <0.001, after multiplicity correction using the Benjamini-Hochberg procedure.

**Figure 4:**
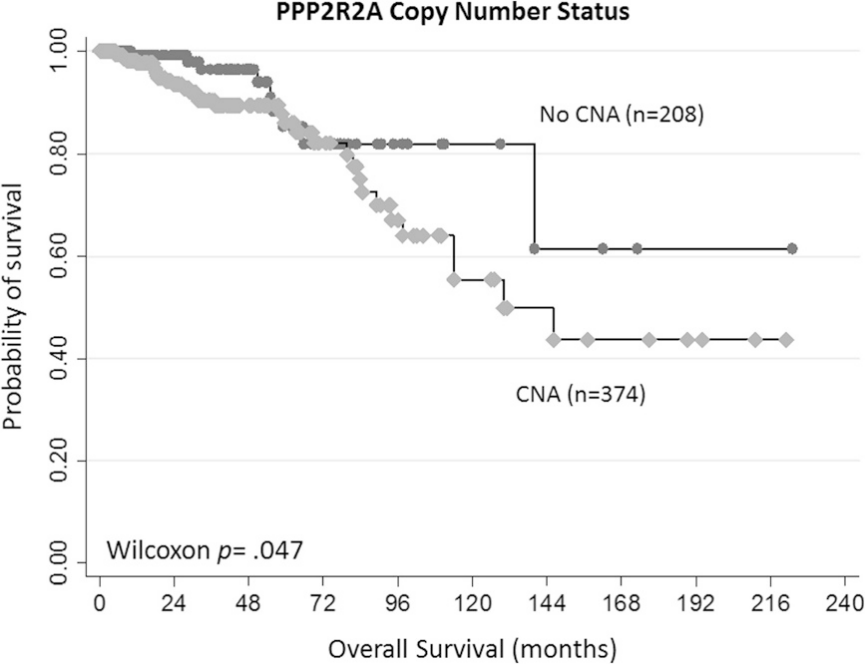
Survival probability according to CNA on PPP2R2A loci. In the TCGA database, carcinomas harboring CNA (either HetDel or HomDel) at the PPP2R2A locus (n = 374) display significantly shorter OS (Wilcoxon *p* = 0.047).

Using the same dataset (Invasive Breast Cancer dataset, TCGA, provisional), we performed survival analysis on all cases available with CNA data (either HomDel or HetDel) and survival information (n = 582 patients). We compared the OS of patients with CNA (n = 374, 33 reported events) with those without CNA at the PPP2R2A locus (n = 208, 9 reported events) and show that patients with CNA at PPP2R2A have significantly worse survival (Wilcoxon test for equality of survival functions p = 0.047) (Figure 4). Due to the extensive follow-up time and unavailability of the time to progression in the currently publically available version of the dataset, we used the Wilcoxon test because we considered the earlier events to have occurred due to disease recurrence. We performed a sensitivity analysis that supports the assumption that disease-specific events occurred earlier (more details in Additional file 1: Figure S1).

### PPP2R2A (B55α)^-/low^ expression is associated with higher grade and ER positive carcinomas

In this study, we included 280 tumors from the discovery cohort and 378 from the validation cohort that had PPP2R2A (B55α) expression and outcome data. A summary of the patients’ characteristics is provided in Additional file 1: Table S3 for the discovery cohort and in Additional file 1: Table S4 for the validation cohort. The prevalence of carcinomas with PPP2R2A (B55α) ^-/**low**^ expression was similar in both cohorts, with 51.8% (n = 145) and 54.0% (n = 204) of tumors expressing PPP2R2A (B55α) in the discovery and validation cohorts, respectively. In the discovery cohort, tumors displaying PPP2R2A (B55α) ^-/**low**^ expression are associated with ER expression (*p* < 0.001), higher grade (*p* = 0.004) and Her2 overexpression (*p* < 0.001). Tumors displaying PPP2R2A (B55α)^-/**low**^ expression are also associated with shortened DFS and OS (log-rank *p* < 0.001 and *p* = 0.002, respectively) (Additional file 1: Table S3). Associations between PPP2R2A (B55α) ^-/**low**^ expression and ER expression and short DFS and OS were further confirmed in the validation cohort (*p* = 0.001 for ER; log-rank *p* = 0.006 for DFS and *p* =0.033 for OS) (Additional file 1: Table S4).

Due to the hypothesis set *a priori* regarding the association between PPP2R2A (B55α) expression and ER status, which was subsequently tested on the two independent cohorts used, we conducted further analysis restricted to the luminal-like BC subgroup.

### PPP2R2A (B55α) expression defines luminal-like BC groups with distinctive outcomes

**Figure 5:**
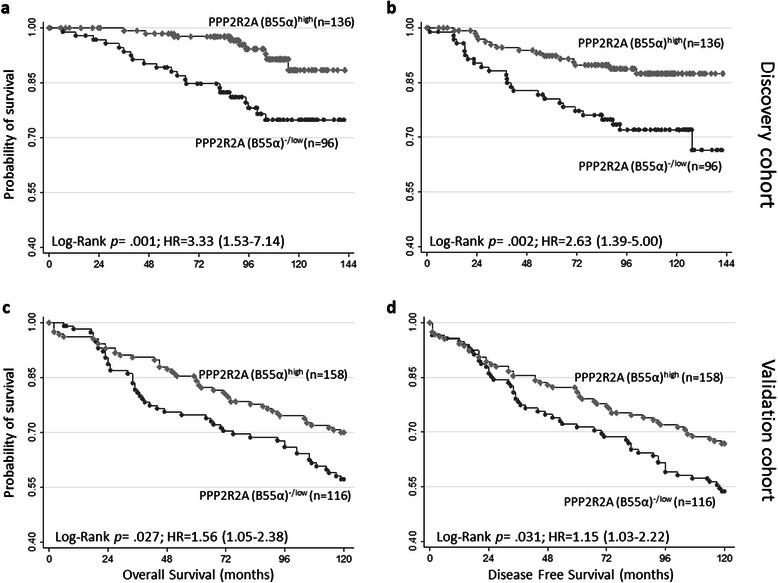
Survival probability according to PPP2R2A (B55α) expression status. PPP2R2A (B55α)^-/low^ carcinomas are associated with lower survival probability: **(a)** OS probability in luminal BC patients of the discovery cohort (n = 232); **(b)** DFS probability in luminal BC patients of the discovery cohort (n = 232); **(c)** OS probability in luminal BC patients of the validation cohort (n = 274); **(d)** DFS probability in luminal BC patients of the validation cohort (n = 274).

When restricting the analysis to the luminal-like BC subgroup, in the discovery cohort, 41.4% (n = 96) of the cases display PPP2R2A (B55α) ^-/low^ expression, while in the validation cohort, these tumors correspond to 42.3% (n = 116) of luminal BC (Additional file 1: Table S5 and Additional file 1: Table S6, respectively). Again, significant associations with grade and PPP2R2A (B55α) expression status are found, with PPP2R2A (B55α)^-/low^ tumors displaying higher grade (*p* = 0.004). Luminal BCs with PPP2R2A (B55α) ^-/low^ are also associated with a higher prevalence of Her-2 overexpression (*p* < 0.001), although no association with the Ki-67 proliferation index was found (*p* = n.s.) (Additional file 1: Table S5). PPP2R2A (B55α) ^-/ low^ expression status also defined subgroups of luminal BCs with differential outcomes, with PPP2R2A (B55α) ^-/ low^-expressing BC associating with worse DFS and OS (log-rank: *p* = 0.002 and *p* = 0.001, respectively (Figure 5). Despite considerable differences in the baseline characteristics between luminal-like cases in the discovery and validation cohorts, namely regarding age, tumor size and grade (Additional file 1: Table S7), the association between PPP2R2A (B55α) expression status and worse DFS and OS was further confirmed in this subset of patients in the validation cohort with PPP2R2A (B55α)^-/ low^ BC (*p* = 0.031 for DFS and *p* = 0.027 for OS). In the luminal-like BC subgroup, in both cohorts, PPP2R2A (B55α)^-/ low^ is significantly associated with steeper mortality curves, thus further validating the scoring methodology used and the association between PPP2R2A (B55α) expression status and survival in this subset of patients (Figure 5).

### PPP2R2A (B55α) ^-/low^ /Cyclin D1^high^ expression defines a group of luminal BC with worse outcomes

**Figure 6:**
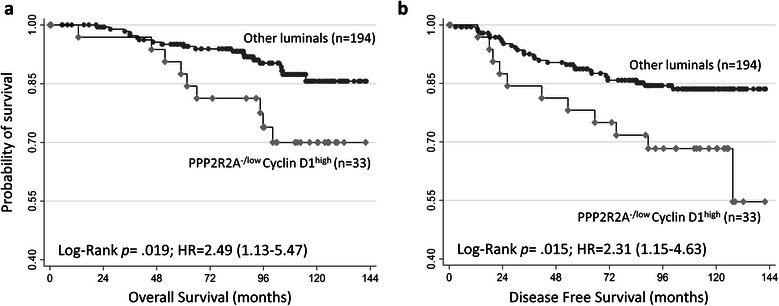
Survival probability according to the phenotype defined by PPP2R2A (B55α) and Cyclin D1 expression status. Irrespective of other clinicopathological features, in the luminal BC type, the PPP2R2A (B55α) ^-/low^ and Cyclin D1^high^ phenotype associates with worse OS **(a)** and worse DFS **(b)** (n = 227).

To address the secondary hypothesis that the combination of altered PPP2R2A (B55α) protein expression and Cyclin D1 expression would define a group of luminal BC with worse outcomes, we first evaluated Cyclin D1 expression in the luminal BC cases of the discovery cohort. In this cohort, the overexpression of Cyclin D1 is associated with higher grade carcinomas (*p* = 0.029), but there was no significant association with any of the survival outcomes (Additional file 1: Figure S2). Next, we selected the cases with Cyclin D1 overexpression from those with negative/low expression of PPP2R2A (B55α), thus defining the phenotype PPP2R2A^-/low^/Cyclin D1^high^. The prevalence of this phenotype is 11.9% in the complete discovery cohort and 14.5% when restricting luminal-like BC (Table 1). Interestingly, despite the association with higher grade (*p* = 0.014) and Her-2 OE carcinomas (*p* = 0.020), 51.5% of the cases displaying the phenotype PPP2R2A^-/low^/Cyclin D1^high^ would be otherwise classifiable as luminal A-like (Table 1) and the presence of the phenotype was also associated with both worse OS (HR 2.71, 95% CI 1.17–6.28, *p* = 0.020) and DFS (HR 2.63, 95% CI 1.26–5.11, *p* = 0.010) (Table 1 & Figure 6). Since this effect on survival could be largely dependent on HER2 status, disease stage and proliferation index, we performed multivariate Cox proportional hazards survival analysis adjusting for HER2 status, tumor size and lymph node status, after performing multiple imputation of missing values for lymph node status (more information on modeling is provided in the Additional file 1). The proliferation index variable was dropped due to a lack of significance in the univariate analysis using the set cut-off of 14% (HR 1.88, 95% CI 0.84–4.19, *p* = 0.120 for OS; Additional file 1: Figure S3). In this multivariate model for PPP2R2A^-/low^/Cyclin D1^high^ cases, the HR for OS is 2.09 (95% CI 0.89–4.96, *p* = 0.091), and the HR for DFS is 2.18 (95% CI 1.02–4.66, *p* = 0.043) (Table 2). Thus, in luminal-like BC, the PPP2R2A^-/low^/Cyclin D1^high^ phenotype is significantly associated with worse DFS, independent of HER2 status, tumor size and lymph node status (Table 2).

**Table 1:** **Discovery cohort - Clinico-pathological data of luminal-like BC patients**

Parameter	Status	Total n=227	PPP2R2A (B55α)^-/low^Cyclin D1^high^n=33 (%)	Others n=194 (%)	*p*
Age	≤ 50	91	14 (15.38)	77 (84.62)	.767
	> 50	136	19 (13.97)	117 (86.03)	
	n/a	-			
Size	≤ 2 cm	120	18 (15.00)	102 (85.00)	.834
	> 2 cm	107	15 (14.02)	92 (85.98)	
	n/a	-			
Grade	I	63	3 (4.76)	60 (95.24)	.014
	II	108	17 (15.74)	91 (84.26)	
	III	55	13 (23.64)	42 (76.36)	
	n/a	1		1	
Lymph node status	LNN	105	14 (13.33)	91 (86.67)	.306
	Node pos.	103	18 (17.48)	85 (82.52)	
	n/a	19	1	18	
PgR	Positive	90	11 (12.22)	79 (87.78)	.615*
	Negative	10	2 (20.00)	8 (80.00)	
	n/a	127	78	51	
HER2	Positive	29	9 (31.03)	20 (68.97)	.020*
	Negative	198	24 (12.12)	174 (87.88)	
	n/a	-			
KI-67 Index	<14	152	18 (11.84)	134 (88.16)	.125
	≥14	60	12 (20.00)	48 (80.00)	
	n/a	15	3	12	
Luminal subtype	A-like	152	17 (11.18)	135 (88.82)	.041
	B-like	75	16 (21.33)	59 (78.67)	
	n/a	-			
DFS	Num. of events	41	15	26	.002^#^
	Mean (months)	91	92	89	
OS	Num. of events	30	9	21	.001^#^
	Mean (months)	95	95	95	

Abbreviations: LNN, Lymph node negative; PgR, Progesterone Receptor; HER2, human epidermal growth factor receptor-2.

*Fisher exact test p-value. # Log-Rank test p-value.

**Table 2:** **Univariate and multivariate hazard ratios for OS in luminal breast cancer patients (n=211)**

		OS				DFS			
		Univariate		Multivariate		Univariate		Multivariate	
Variable		HR (95% CI)	p*	HR (95% CI)	p*	HR (95% CI)	p*	HR (95% CI)	p*
PPP2R2A (B55α)^-/low^ Cyclin D1^high^	Present vs. Absent	2.71(1.17-6.28)	.020	2.09(.89-4.96)	.091	2.63(1.26-5.11)	.010	2.18(1.02-4.66)	.043
HER2	Positive vs. Negative	3.37(1.41-8.08)	.006	3.94(1.27-7.55)	.013	3.02(1.37-6.68)	.006	2.92(1.29-6.55)	.010
Size	>2cm vs.<2cm	5.19(1.95-13.85)	.001	3.64(1.39-11.18)	.010	6.00(2.48-14.51)	<.001	4.47(1.91-2.14)	.001
Lymph node status^#^	LNN vs. Positive	4.13(1.47-11.58)	.007	2.49(0.84-7.34)	.099	4.08(1.71-9.72)	.002	2.36(.99-5.64)	.052
Ki-67 index	>14% vs.≥14%	1.88(.84-4.19)	.12	-	-	2.46(1.25-4.83)	.009	-	-

Abbreviations: LNN, Lymph node negative; PgR, Progesterone Receptor; HER2, human epidermal growth factor receptor-2.

*Cox-proportional hazards model. ^#^Results obtained after using multiple imputation to deal with the 17 cases with missing values for Nodal disease. Nodal disease values were imputed adjusting for tumor size, the PPP2R2A (B55α)/Cyclin D1 phenotype and HER2 status.

## Discussion

Currently, the PP2A holoenzyme complex and certain subunits including PPP2R2A (B55α) are thought to be tumor suppressors in many cancer types [6,9,25,26]. However, the expression status of the PP2A holoenzyme complex subunits, specifically PPP2R2A, as a biomarker has hardly been explored in any cancer type, including BC. Here, we present the first study to evaluate PPP2R2A (B55α) expression in BC and show that CNA, namely deletions centered on PPP2R2A in the most recent TCGA Breast Cancer dataset, is associated with worse OS. We also provide pioneering evidence that absence of PPP2R2A (B55α) expression in BC is associated with an aggressive phenotype, even in strikingly different cohorts of patients.

In contrast to the lack of data on PPP2R2A (B55α), there are numerous publications on the prognostic and predictive roles of Cyclin D1 overexpression and CCND1 amplification, which are usually associated with early relapse and poor prognosis in ER-positive BC [20,27-31]. Taking in account some of the reported characteristics of the Integrative Cluster 2 BC [9,12], we hypothesized that using a combined analysis of Cyclin D1 and PPP2R2A (B55α) expression assessed by IHC, we would be able to identify an immunophenotype of luminal BC that is associated with poor OS and DFS. Indeed, we show that in luminal BC, the PPP2R2A^-/low^/Cyclin D1^high^ phenotype is associated with higher histological grade and concomitant HER-2 overexpression, as well as shorter DFS and OS. Moreover, approximately half of the cases with the PPP2R2A^-/low^/Cyclin D1^high^ phenotype correspond to luminal B-like BC, which is similar to the prevalence of luminal B BC in Integrative Cluster 2 BC [12]. However, we also show that the association with worse DFS is independent of HER2-status, as well as tumor size and lymph node status. Thus, we provide additional clinicopathological support for the role of PPP2R2A (B55α) as a tumor suppressor in BC and present pilot evidence that the PPP2R2A^-/low^/Cyclin D1^high^ phenotype may be clinically relevant in BC and should be further investigated.

We consider the PPP2R2A (B55α) scoring methodology proposed in our study to be fairly robust, as both cohorts had similar proportions of cases assigned to the various groups. Moreover, given the clinicopathological differences between the cohorts, the PPP2R2A (B55α) scoring methodology was able to define groups displaying comparable magnitudes of HR, thus further reinforcing the reproducibility of the method. Regarding Cyclin D1 evaluation, we are confident in the validity of the due to the extensive validation of the IHC methodologies in the literature [20,28,32,33]. However, some important limitations remain. Although we did not directly demonstrate the loss of B55α immunoreactivity in cases with known deletions of the chr 8p21.2 region, we show that in the TCGA Invasive Breast Cancer Dataset there is a strong correlation between CNA and PPP2R2A (B55α) mRNA expression in 736 BCs. Interestingly, in luminal-like BC and other subtypes, multiple mechanisms could contribute to PPP2R2A (B55α) downregulation. In fact, recent reports have shown that in BC, two inhibitors of PP2A, SET and CIP2A are frequently overexpressed in triple negative, basal and claudin-low subtypes [10]. Therefore, because PPP2R2A (B55α) expression detected by IHC reflects not only CNA, it could be a highly useful biomarker in BC, including luminal-like BC. Despite controlling for major determinants of BC survival, we were unable to determine if this marker was prognostic or predictive because we did not study the possible interactions between this phenotype and treatment variables. Nonetheless, we achieved our objective of determining if it is possible to define an aggressive subgroup of luminal-like BC based exclusively on the phenotype assessed by IHC.

## Conclusion

In conclusion, the results of this study show that CNAs in the PPP2R2A gene and its expression are associated with worse OS and that the PPP2R2A^-/low^/Cyclin D1^high^ phenotype is associated with aggressiveness in luminal-like BC. Importantly, not only does the PPP2R2A^-/low^/Cyclin D1^high^ phenotype add prognostic information to otherwise HER2-expressing luminal BC, it also allows the identification of a subgroup of luminal BC with reduced DFS that would be otherwise classified as luminal A-like. Consequently, these results provide one of the first indications for the clinical relevance of this phenotype in human BC. Based on the functional characteristics and cancer-specific expression pattern of PPP2R2A (B55α) and Cyclin D1, it is evident that both PPP2R2A and the PPP2R2A^-/low^/Cyclin D1^high^ phenotype deserve further attention to better uncover their possible clinical and biological importance in BC.

## Additional files

Additional file 1: Supplementary Data (SD) - Supplementary Methods: Clinical definitions of the survival end-points, Tissue Microarray (TMA) construction and ER, PgR, HER2 and Ki-67 IHC, SD Table 1 - Antibodies used in the immunohistochemistry study, PPP2R2A (B55α) and Cyclin D1 IHC, PPP2R2A (B55α) expression evaluation and cut-off determination, SD Table 2 - Cut-off determination using minimum p-value; Supplementary Results: SD Table 3 – Discovery cohort: Clinico-pathological data of the patients, SD Table 4 – Validation cohort: Clinico-pathological data of the patients, SD Table 5 – Discovery cohort: Clinico- pathological data of Luminal BC patients, SD Table 6 – Validation cohort: Clinico-pathological data of Luminal BC patients, SD Table 7 – Comparison of the clinico-pathological characteristic of the cohorts, SD Figure 1 – Survival probability according to copy number status on PPP2R2A and sensitivity analysis, SD Figure 2 – Survival probability according to Cyclin D1 (CCND1) expression status, SD Figure 3–Forrest plot with odds-ratio for predictors of OS, Survival Model of PPP2R2A^-/low^/Cyclin D1^high^ phenotype; Supplementary Data References.
Additional file 2: STROBE Statement—checklist of items that should be included in reports of observational studies.

## Abbreviations

AML: Acute myeloid leukemia
ASCO: American Society of Clinical Oncology
BC: Breast carcinoma
CAP: College of American Pathologists
CI: Confidence interval
CNA: Copy number alteration
DFS: Disease free survival
ER: Estrogen receptor
Her 2: Human epidermal growth factor receptor 2
HR: Hazard ratio
H&E: Hematoxylin and eosin
IHC: Immunohistochemistry
LN: Lymph node
OE: Overexpressing
OS: Overall survival
PgR: Progesterone receptor
PP2A: Protein phosphatase 2
REMARK: Reporting recommendations for tumour marker prognostic studies
SD: Supplementary data
STROBE: Strengthening the reporting of observational studies in Epidemiology
TCGA: Cancer genome atlas
TMAs: Tissue microarrays

## References

[1] Mumby M (2007). The 3D structure of protein phosphatase 2A: new insights into a ubiquitous regulator of cell signaling. ACS Chem Biol.

[2] Kurimchak A, Grana X (2012). PP2A holoenzymes negatively and positively regulate cell cycle progression by dephosphorylating pocket proteins and multiple CDK substrates. Gene.

[3] Shi Y (2009). Serine/threonine phosphatases: mechanism through structure. Cell.

[4] Suganuma M, Fujiki H, Furuya-Suguri H, Yoshizawa S, Yasumoto S, Kato Y (1990). Calyculin A, an inhibitor of protein phosphatases, a potent tumor promoter on CD-1 mouse skin. Cancer Res.

[5] Pallas DC, Shahrik LK, Martin BL, Jaspers S, Miller TB, Brautigan DL (1990). Polyoma small and middle T antigens and SV40 small t antigen form stable complexes with protein phosphatase 2A. Cell.

[6] Cristobal I, Manso R, Rincon R, Carames C, Senin C, Borrero A (2014). PP2A inhibition is a common event in colorectal cancer and its restoration using FTY720 shows promising therapeutic potential. Mol Cancer Ther.

[7] Wang Y, Waters J, Leung ML, Unruh A, Roh W, Shi X (2014). Clonal evolution in breast cancer revealed by single nucleus genome sequencing. Nature.

[8] Kalev P, Simicek M, Vazquez I, Munck S, Chen L, Soin T (2012). Loss of PPP2R2A inhibits homologous recombination DNA repair and predicts tumor sensitivity to PARP inhibition. Cancer Res.

[9] Ruvolo PP, Qui YH, Coombes KR, Zhang N, Ruvolo VR, Borthakur G (2011). Low expression of PP2A regulatory subunit B55alpha is associated with T308 phosphorylation of AKT and shorter complete remission duration in acute myeloid leukemia patients. Leukemia.

[10] Janghorban M, Farrell AS, Allen-Petersen BL, Pelz C, Daniel CJ, Oddo J (2014). Targeting c-MYC by antagonizing PP2A inhibitors in breast cancer. Proc Natl Acad Sci U S A.

[11] Dupont WD, Breyer JP, Bradley KM, Schuyler PA, Plummer WD, Sanders ME (2010). Protein phosphatase 2A subunit gene haplotypes and proliferative breast disease modify breast cancer risk. Cancer.

[12] Curtis C, Shah SP, Chin SF, Turashvili G, Rueda OM, Dunning MJ (2012). The genomic and transcriptomic architecture of 2,000 breast tumours reveals novel subgroups. Nature.

[13] Cerami E, Gao J, Dogrusoz U, Gross BE, Sumer SO, Aksoy BA (2012). The cBio cancer genomics portal: an open platform for exploring multidimensional cancer genomics data. Canc Discov.

[14] Gao J, Aksoy BA, Dogrusoz U, Dresdner G, Gross B, Sumer SO (2013). Integrative analysis of complex cancer genomics and clinical profiles using the cBioPortal. Sci Signal.

[15] Elston CW, Ellis IO (1991). Pathological prognostic factors in breast cancer. Histopathology.

[16] de Beca FF, Caetano P, Gerhard R, Alvarenga CA, Gomes M, Paredes J (2013). Cancer stem cells markers CD44, CD24 and ALDH1 in breast cancer special histological types. J Clin Pathol.

[17] Goldhirsch A, Winer EP, Coates AS, Gelber RD, Piccart-Gebhart M, Thurlimann B (2013). Panel m:Personalizing the treatment of women with early breast cancer: highlights of the St Gallen International Expert Consensus on the Primary Therapy of Early Breast Cancer 2013. Ann Oncol.

[18] Cheang MC, Chia SK, Voduc D, Gao D, Leung S, Snider J (2009). Ki67 index, HER2 status, and prognosis of patients with luminal B breast cancer. J Natl Cancer Inst.

[19] Wolff AC, Hammond ME, Hicks DG, Dowsett M, McShane LM, Allison KH (2013). Recommendations for human epidermal growth factor receptor 2 testing in breast cancer: American Society of Clinical Oncology/College of American Pathologists clinical practice guideline update. J Clin Oncol.

[20] Roy PG, Pratt N, Purdie CA, Baker L, Ashfield A, Quinlan P (2010). High CCND1 amplification identifies a group of poor prognosis women with estrogen receptor positive breast cancer. Int J Canc Suppl J Int Canc Suppl.

[21] Martins D, Beca FF, Sousa B, Baltazar F, Paredes J, Schmitt F (2013). Loss of caveolin-1 and gain of MCT4 expression in the tumor stroma: key events in the progression from an in situ to an invasive breast carcinoma. Cell Cycle.

[22] Mazumdar M, Glassman JR (2000). Categorizing a prognostic variable: review of methods, code for easy implementation and applications to decision-making about cancer treatments. Stat Med.

[23] McShane LM, Altman DG, Sauerbrei W, Taube SE, Gion M, Clark GM (2006). REporting recommendations for tumor marker prognostic studies (REMARK). Breast Cancer Res Treat.

[24] von Elm E, Altman DG, Egger M, Pocock SJ, Gotzsche PC, Vandenbroucke JP (2007). The Strengthening the Reporting of Observational Studies in Epidemiology (STROBE) statement: guidelines for reporting observational studies. Lancet.

[25] Schmitz MH, Held M, Janssens V, Hutchins JR, Hudecz O, Ivanova E (2010). Live-cell imaging RNAi screen identifies PP2A-B55alpha and importin-beta1 as key mitotic exit regulators in human cells. Nat Cell Biol.

[26] Alvarez-Fernandez M, Halim VA, Aprelia M, Laoukili J, Mohammed S, Medema RH (2011). Protein phosphatase 2A (B55alpha) prevents premature activation of forkhead transcription factor FoxM1 by antagonizing cyclin A/cyclin-dependent kinase-mediated phosphorylation. J Biol Chem.

[27] Cuny M, Kramar A, Courjal F, Johannsdottir V, Iacopetta B, Fontaine H (2000). Relating genotype and phenotype in breast cancer: an analysis of the prognostic significance of amplification at eight different genes or loci and of p53 mutations. Cancer Res.

[28] Elsheikh S, Green AR, Aleskandarany MA, Grainge M, Paish CE, Lambros MB (2008). CCND1 amplification and cyclin D1 expression in breast cancer and their relation with proteomic subgroups and patient outcome. Breast Cancer Res Treat.

[29] Seshadri R, Lee CS, Hui R, McCaul K, Horsfall DJ, Sutherland RL (1996). Cyclin DI amplification is not associated with reduced overall survival in primary breast cancer but may predict early relapse in patients with features of good prognosis. Clin Canc Res.

[30] Michalides R, Hageman P, van Tinteren H, Houben L, Wientjens E, Klompmaker R (1996). A clinicopathological study on overexpression of cyclin D1 and of p53 in a series of 248 patients with operable breast cancer. Br J Cancer.

[31] Bostner J, Ahnstrom Waltersson M, Fornander T, Skoog L, Nordenskjold B, Stal O (2007). Amplification of CCND1 and PAK1 as predictors of recurrence and tamoxifen resistance in postmenopausal breast cancer. Oncogene.

[32] Jirstrom K, Stendahl M, Ryden L, Kronblad A, Bendahl PO, Stal O (2005). Adverse effect of adjuvant tamoxifen in premenopausal breast cancer with cyclin D1 gene amplification. Cancer Res.

[33] Mrhalova M, Kodet R, Strnad P (2002). [Invasive ductal carcinoma of the breast: study of the number of copies of the CCND1 gene and chromosome 11 using fluorescence in situ hybridization (FISH) in comparison with expression of cyclin D1 protein and estrogen receptors (ER alpha) with immunohistochemical detection]. Cas Lek Cesk.

