# Supplementary material for: Altered PPP2R2A and Cyclin D1 expression defines a subgroup of aggressive luminal-like breast cancer
Source: BMC Cancer. 2015 Apr 15;15:285. doi: 10.1186/s12885-015-1266-1 (PMC4409761; doi:10.1186/s12885-015-1266-1)
Supplement: Additional file 1: — Supplementary Data (SD) - Supplementary Methods: Clinical definitions of the survival end-points, Tissue Microarray (TMA) construction and ER, PgR, HER2 and Ki-67 IHC, SD Table 1 - Antibodies used in the immunohistochemistry study, PPP2R2A (B55α) and Cyclin D1 IHC, PPP2R2A (B55α) expression evaluation and cut-off determination, SD Table 2 - Cut-off determination using minimum p-value; Supplementary Results: SD Table 3 – Discovery cohort: Clinico-pathological data of the patients, SD Table 4 – Validation cohort: Clinico-pathological data of the patients, SD Table 5 – Discovery cohort: Clinico- pathological data of Luminal BC patients, SD Table 6 – Validation cohort: Clinico-pathological data of Luminal BC patients, SD Table 7 – Comparison of the clinico-pathological characteristic of the cohorts, SD Figure 1 – Survival probability according to copy number status on PPP2R2A and sensitivity analysis, SD Figure 2 – Survival probability according to Cyclin D1 (CCND1) expression status, SD Figure 3–Forrest plot with odds-ratio for predictors of OS, Survival Model of PPP2R2A-/low/Cyclin D1high phenotype; Supplementary Data References. [file 12885_2015_1266_MOESM1_ESM.docx]

**Supplementary Data (SD)**

Supplementary Methods 1

Clinical definitions of the survival end-points 1

Tissue Microarray (TMA) construction and ER, PgR, HER2 and Ki-67 IHC 1

SD Table 1 - Antibodies used in the immunohistochemistry study 2

PPP2R2A (B55α) and Cyclin D1 IHC 2

PPP2R2A (B55α) expression evaluation and cut-off determination 2

SD Table 2 - Cut-off determination using minimum p-value 3

Supplementary Results 4

SD Table 3 – Discovery cohort: Clinico-pathological data of the patients 4

SD Table 4 – Validation cohort: Clinico-pathological data of the patients 5

SD Table 5 – Discovery cohort: Clinico-pathological data of Luminal BC patients 6

SD Table 6 – Validation cohort: Clinico-pathological data of Luminal BC patients 7

SD Table 7 – Comparison of the clinico-pathological characteristic of the cohorts 7

SD Figure 1 – Survival probability according to copy number status on PPP2R2A and sensitivity analysis 8

SD Figure 2 – Survival probability according to Cyclin D1 (CCND1) expression status. 8

SD Figure 3 – Forrest plot with odds-ratio for predictors of OS 9

Survival Model of PPP2R2A^-/low^/Cyclin D1^high^ phenotype 9

Supplementary Data References 10

# Supplementary Methods

## Clinical definitions of the survival end-points

**Overall survival (OS)** time was defined as the time between the diagnosis date and the documented date of death.

**Disease-free survival (DFS)** was defined as the time between the diagnosis date and the first locoregional or systemic relapse date. Local relapse was defined as the reappearance of carcinoma in the treated remnant breast, skin, or chest wall. Events determining regional relapse were defined as tumor recurrences in the ipsilateral axillary or internal mammary lymph nodes. Recurrence at a distant site, including the contralateral axillary or supraclavicular lymph nodes, was considered to be a distant metastasis.

## Tissue Microarray (TMA) construction and ER, PgR, HER2 and Ki-67 IHC

Representative areas of the invasive breast carcinomas were selected on H&E stained sections and marked on the correspondent individual paraffin block. Two tissue cores (2mm in diameter) were obtained from each specimen for TMA construction, with each TMA block (donor block) and deposited into a paraffin block (receptor block), using a TMA workstation (TMA builder ab1802, Abcam, Cambridge, UK). Each TMA block constructed, contained 24 tissue cores, in a 4x6 arrangement. In each TMA block, non-neoplastic breast and testis tissue cores were also included as controls and TMA guides, respectively. An H&E stained section from each TMA block was reviewed to confirm the presence of morphological representative areas of the original lesions.

As information regarding estrogen receptor (ER), progesterone receptor (PgR) and HER-2 was available from the anatomic pathology reports of the discovery cohort, immunohistochemistry was performed for antigen Ki-67 in cases missing that information. For the validation cohort, ER, PgR, HER-2 and Ki-67 was performed. HER-2 expression was evaluated according to the DakoCytomation Hercept Test® scoring system for both cohorts [[1](#_ENREF_1)]. Cases were considered positive (overexpression) for HER-2 when immunostaining was classified as 3+. In cases classified as 2+ by immunohistochemistry (IHC), FISH (fluorescence in situ hybridization) analysis was performed to assess HER2 gene amplification. Tumors were classified as luminal-like if presented positivity for hormone receptor (ER and PgR). Cases lacking ER or PgR with HER-2 overexpression were classified as HER-2 overexpressing tumors. ER, PgR and HER-2 negative were classified as triple-negative (TNBC).

| **SD Table 1** - Antibodies used for IHC | | | | | | |
| --- | --- | --- | --- | --- | --- | --- |
| **Antigen** | **Source** | **Clone** | **Dilution** | **Incubation** | **Antigen retrieval** | **Detection reagent** |
| **ER** | Neomarkers | SP1 | 1:100 | 1 hr RT | Citrate buffer, pH 6.0 30min at 98ºC | HRP polymer |
| **PgR** | Novocasta | 1A6 | 1:40 | 1 hr RT | Citrate buffer, pH 6.0 30min at 98ºC | HRP polymer |
| **HER2** | Neomarkers | SP3 | 1:80 | 30 min RT | Citrate buffer, pH 6.0 30min at 98ºC | SABC |
| **Ki-67** | Neomarkers | SP6 | 1:200 | 1 hr RT | Citrate buffer, pH 6.0 30min at 98ºC | HRP polymer |

RT – Room Temperature; HRP – Horseradish peroxidase; SABC – Streptavidin‐biotin complex

## PPP2R2A (B55α) and Cyclin D1 IHC

IHC was performed using the HRP labeled polymer (DakoCytomation, Carpinteria,CA, USA) for PPP2R2A (B55α) and Cyclin D1. Antigen unmasking for PPP2R2A (B55α) was carried out using tris-ethylenediaminetetraacetic (EDTA) solution (pH=9.0) (DakoCytomation), whereas a commercially available solution of citrate buffer, pH=6.0 (Vector Laboratories, Burlingame, CA, USA) at 98ºC was used for Cyclin D1.

After the antigen retrieval procedure, the slides were washed in a phosphate buffer solution (PBS) and submitted to blockage of the endogenous peroxidase activity by incubation of the slides in a 3% hydrogen peroxide (Panreac, Spain) in methanol (Sigma-Aldrich). The slides were further incubated with the primary antibodies for PPP2R (B55α) (sc-81606; Santa Cruz Biotecnhology, diluted 1:50) and for Cyclin D1 (SP4; Neomarkers, diluted 1:50). All reactions were revealed with diaminobenzidine (DAB) chromogen (DakoCytomation).

For both IHC assays, positive controls were included in each run, in order to guarantee the reliability of the assays.

## PPP2R2A (B55α) expression evaluation and cut-off determination

Due to external validity concerns and since to the best of our knowledge there is no published data regarding a PPP2R2A (B55α) scoring methodology in breast carcinoma, we used two different independent cohorts: we first used a cohort to establish the IHC ideal assessment criteria for PPP2R2A (B55α) expression (Discovery cohort) and posteriorly applied the exact same methodology in an independent cohort to test the validity of the methodology used (Validation cohort).

PPP2R2A (B55α) IHC expression was initially annotated semi-quantitatively in the cytoplasm of invasive carcinoma in the discovery cohort using the previously defined scoring method[[2](#_ENREF_2), [3](#_ENREF_3)]. Briefly, sections were scored semi-quantitatively as follows: 0, 0-1% immuno-reactive cells; 1, < 5% immuno-reactive cells; 2, 5–50% immuno-reactive cells; and 3, > 50% immuno-reactive cells. Similarly, intensity of staining was evaluated semi-quantitatively on a scale from 0 to 3, with 0 representing negative; 1, weak; 2, moderate and 3, strong staining. The final score was calculated, by adding extension to intensity scores and ranged between 0 and 6 points. Afterwards, using the discovery cohort, we determined the ideal cut-off for dichotomization of the PPP2R2A (B55α) scoring using the minimum p-value method on log-rank test for overall survival[[4](#_ENREF_4)]. Based on our biological rational and our objective to detect complete or nearly complete loss of expression of PPP2R2A (B55α), we started by testing possible cut-off points on the lower end of the scoring scale as they would be more stringent and biologically plausible. The following table illustrates the results of this approach:

| **SD Table 2 -** Cut-off determination using minimum p-value | | | | | |
| --- | --- | --- | --- | --- | --- |
| PPP2R2A (B55α) IHC score | P-value on log-rank test | |  | Prevalence of PPP2R2A (B55α)^-/low^ | |
|  | Complete cohort (n=280) | Luminal-like BC  (n=232) |  | Complete cohort (n=280) | Luminal-like BC  (n=232) |
| 2 | 0.0089 | 0.0029 |  | 45.96 (129) | 40.25 (93) |
| 3 | 0.0024 | 0.0011 |  | 48.77 (137) | 41.37 (96) |
| 4 | 0.0006 | 0.0012 |  | 68.42 (192) | 62.29 (145) |

Since the score 3 dichotomized the PPP2R2A (B55α) IHC expression with lower p-value for OS in the luminal-like BC (with a low p-value also on the complete cohort), and a number of cases similar to lower scores (more stringent to define negative/low expression) we used the score 3 as a cut-off point of PPP2R2A (B55α) IHC expression. Briefly, cases with a score (derived from extension and intensity) lower than 3 were classified as PPP2R2A (B55α)^-/low^, corresponding to: 1) no expression or 2) weak expression in less than 5% of cells. All other cases, displaying moderate or high expression in any extension or weak expression in more than 5% of cells were classified as positive for PPP2R2A (B55α) expression (PPP2R2A (B55α)^high^).

# Supplementary Results

| SD Table 3 – Discovery cohort: Clinico-pathological data of the patients | | | | | |
| --- | --- | --- | --- | --- | --- |
| **Parameter** | **Status** | **Total**  **n=280** | **PPP2R2A (B55α) expression** | | ***p*** |
|  |  |  | **High (n=145)** | **-/low (n=186)** |  |
| Age | ≤ 50 | 114 | 56 (49.12) | 58 (50.88) | 0.460 |
|  | > 50 | 166 | 89 (53.61) | 77 (46.39) |  |
|  | n/a | - |  |  |  |
| Size | ≤ 2 cm | 144 | 82 (56.94) | 62 (43.06) | 0.075 |
|  | > 2 cm | 136 | 63 (46.32) | 73 (53.68) |  |
|  | n/a | - |  |  |  |
| Grade | I | 64 | 45 (70.31) | 19 (29.69) | <0.001 |
|  | II | 119 | 69 (57.98) | 50 (42.02) |  |
|  | III | 96 | 30 (31.25) | 66 (68.75) |  |
|  | n/a | 1 | 1 |  |  |
| Lymph node status | LNN | 129 | 72 (55.81) | 57 (44.19) | 0.121 |
|  | Node pos. | 132 | 61 (46.21) | 71 (53.79) |  |
|  | n/a | 19 | 12 | 7 |  |
| ER | Positive | 232 | 136 (58.62) | 96 (41.38) | <0.001 |
|  | Negative | 48 | 9 (18.75) | 39 (81.25) |  |
|  | n/a | - |  |  |  |
| PgR | Positive | 94 | 53 (56.38) | 41 (43.62) | <0.001 |
|  | Negative | 53 | 14 (26.42) | 39 (73.58) |  |
|  | n/a | 133 | 78 | 55 |  |
| HER2 | Positive | 55 | 11 (20.00) | 44 (80.00) | <0.001 |
|  | Negative | 225 | 134 (59.56) | 91 (40.44) |  |
|  | n/a | - |  |  |  |
| KI-67 Index | <14 | 171 | 98 (57.31) | 73 (42.69) | 0.017 |
|  | ≥14 | 93 | 39 (41.94) | 54 (58.06) |  |
|  | n/a | 52 | 9 | 12 |  |
| Molecular Subtype | Luminal | 232 | 136 (58.62) | 96 (41.38) | <0.001* |
|  | Her2-OE | 24 | 5 (20.83) | 19 (79.17) |  |
|  | TN | 24 | 4 (16.67) | 20 (83.33) |  |
|  | n/a | - |  |  |  |
| Histological Type | IC-NST | 210 | 104 (49.52) | 106 (50.48) | 0.486 |
|  | ILC | 13 | 8 (61.54) | 5 (38.46) |  |
|  | Mixed type IC | 32 | 17 (53.13) | 15 (46.88) |  |
|  | OST | 25 | 16 (64.00) | 9 (36.00) |  |
|  | n/a | - |  |  |  |
| DFS | Num. of events | 58 | 18 | 40 | <0.001^#^ |
|  | Mean (months) | 86 | 90 | 81 |  |
| OS | Num. of events | 38 | 11 | 27 | 0.002^#^ |
|  | Mean (months) | 91 | 93 | 89 |  |
| Abbreviations: LNN, Lymph node negative; ER, estrogen receptor; PgR, Progesterone Receptor; HER2, human epidermal growth factor receptor-2; TN, Triple Negative; IC-NST, Invasive Carcinoma of no special type; ILC, Invasive Lobular Carcinoma; OST, Other Special Type. | | | | | |
| *Fisher exact test p-value. ^#^ Log-Rank test p-value. | | | | | |

| SD Table 4 – Validation cohort: Clinico-pathological data of the patients | | | | | |
| --- | --- | --- | --- | --- | --- |
| **Parameter** | **Status** | **Total**  **n=378** | **PPP2R2A (B55α) expression** | | ***p*** |
|  |  |  | **High (n=204)** | **-/low (n=174)** |  |
| Age | ≤ 50 | 111 | 56 (50.45) | 55 (49.55) | 0.376 |
|  | > 50 | 267 | 148 (55.43) | 119 (44.57) |  |
|  | n/a | - |  |  |  |
| Size | ≤ 2 cm | 140 | 70 (50.00) | 50 (70.00) | 0.235 |
|  | > 2 cm | 238 | 134 (56.30) | 104 (43.70) |  |
|  | n/a | - |  |  |  |
| Grade | I | 70 | 42 (60.00) | 28 (40.00) | 0.494 |
|  | II | 109 | 57 (52.29) | 52 (47.71) |  |
|  | III | 194 | 101 (52.06) | 93 (47.94) |  |
|  | n/a | 5 | 4 | 1 |  |
| Lymph node status | LNN | 156 | 75 (55.15) | 61 (44.85) | 0.821 |
|  | Node pos. | 194 | 101 (56.42) | 78 (43.58) |  |
|  | n/a | 78 | 12 | 7 |  |
| ER | Positive | 246 | 148 (60.16) | 98 (39.84) | 0.001 |
|  | Negative | 131 | 55 (41.98) | 76 (58.02) |  |
|  | n/a | 1 | 1 |  |  |
| PgR | Positive | 184 | 100 (54.35) | 84 (45.65) | 0.849 |
|  | Negative | 193 | 103 (53.37) | 90 (46.63) |  |
|  | n/a | 1 | 1 |  |  |
| HER2 | Positive | 59 | 28 (47.46) | 31 (52.54) | 0.282 |
|  | Negative | 316 | 174 (55.06) | 142 (44.94) |  |
|  | n/a | 3 | 2 | 1 |  |
| Molecular Subtype | Luminal | 274 | 158 (57.66) | 116 (42.34) | 0.057 |
|  | Her2-OE | 29 | 14 (48.28) | 15 (51.72) |  |
|  | TN | 75 | 32 (42.67) | 43 (57.33) |  |
|  | n/a | - |  |  |  |
| DFS | Num. of events | 162 | 75 | 87 | 0.006^#^ |
|  | Mean (months) | 86 | 92 | 80 |  |
| OS | Num. of events | 146 | 70 | 76 | 0.033^#^ |
|  | Mean (months) | 91 | 96 | 85 |  |
| Abbreviations: LNN, Lymph node negative; ER, estrogen receptor; PgR, Progesterone Receptor; HER2, human epidermal growth factor receptor-2; TN, Triple Negative; IC-NST, Invasive Carcinoma of no special type; ILC, Invasive Lobular Carcinoma; OST, Other Special Type. | | | | | |
| ^#^ Log-Rank test p-value. | | | | | |

| SD Table 5 – Discovery cohort: Clinico-pathological data of Luminal BC patients | | | | | |
| --- | --- | --- | --- | --- | --- |
| **Parameter** | **Status** | **Total**  **n=232** | **PPP2R2A (b55α expression)** | | ***p*** |
|  |  |  | **High (n=136)** | **-/low (n=96)** |  |
| Age | ≤ 50 | 94 | 54 (57.45) | 40 (42.55) | 0.764 |
|  | > 50 | 138 | 82 (59.42) | 56 (40.58) |  |
|  | n/a | - |  |  |  |
| Size | ≤ 2 cm | 123 | 82 (66.67) | 44 (35.77) | 0.065 |
|  | > 2 cm | 109 | 57 (52.29) | 52 (47.71) |  |
|  | n/a | - |  |  |  |
| Grade | I | 62 | 44 (70.97) | 18 (29.03) | 0.004 |
|  | II | 113 | 68 (60.18) | 45 (39.82) |  |
|  | III | 56 | 23 (41.07) | 33 (58.93) |  |
|  | n/a | 1 | 1 |  |  |
| Lymph node status | LNN | 108 | 66 (61.11) | 42 (38.89) | 0.306 |
|  | Node pos. | 107 | 58 (54.21) | 49 (45.79) |  |
|  | n/a | 17 | 12 | 5 |  |
| PgR | Positive | 94 | 53 (56.38) | 41 (43.62) | 1.000* |
|  | Negative | 9 | 5 (55.56) | 4 (44.44) |  |
|  | n/a | 129 | 78 | 51 |  |
| HER2 | Positive | 31 | 6 (19.35) | 25 (80.65) | <0.001 |
|  | Negative | 201 | 130 (64.68) | 71 (35.32) |  |
|  | n/a | - |  |  |  |
| KI-67 Index | <14 | 153 | 95 (62.09) | 58 (37.91) | 0.241 |
|  | ≥14 | 60 | 32 (53.33) | 28 (46.67) |  |
|  | n/a | 19 | 9 | 10 |  |
| Molecular Subtype | Luminal A | 155 | 99 (63.87) | 56 (36.13) | 0.021 |
|  | Luminal B (≥14 and/or HER2-OE) | 77 | 37 (48.05) | 40 (51.95) |  |
|  | n/a | - |  |  |  |
| Histological Type | IC-NST | 170 | 97 (57.06) | 73 (42.94) | 0.586* |
|  | ILC | 13 | 8 (61.54) | 5 (38.46) |  |
|  | Mixed type IC | 30 | 17 (56.67) | 13 (43.33) |  |
|  | OST | 19 | 14 (73.68) | 5 (26.32) |  |
|  | n/a | - |  |  |  |
| DFS | Num. of events | 41 | 15 | 26 | 0.002^#^ |
|  | Mean (months) | 91 | 92 | 89 |  |
| OS | Num. of events | 30 | 9 | 21 | 0.001^#^ |
|  | Mean (months) | 95 | 95 | 95 |  |
| Abbreviations: LNN, Lymph node negative; PgR, Progesterone Receptor; HER2-OE, human epidermal growth factor receptor-2 overexpressing; IC-NST, Invasive Carcinoma of no special type; ILC, Invasive Lobular Carcinoma; OST, Other Special Type. | | | | | |
| *Fisher exact test p-value. # Log-Rank test p-value. | | | | | |

| SD Table 6 – Validation cohort: Clinico-pathological data of Luminal BC patients | | | | | |
| --- | --- | --- | --- | --- | --- |
| **Parameter** | **Status** | **Total**  **(n=274)** | **PPP2R2A (B55α expression)** | | ***p*** |
|  |  |  | **High (n=158)** | **-/low (n=116)** |  |
| Age | ≤ 50 | 78 | 41 (52.56) | 37 (47.44) | 0.281 |
|  | > 50 | 196 | 117 (59.69) | 79 (40.31) |  |
|  | n/a | - |  |  |  |
| Size | ≤ 2 cm | 111 | 59 (53.15) | 52 (46.85) | 0.212 |
|  | > 2 cm | 163 | 99 (60.74) | 64 (39.26) |  |
|  | n/a | - |  |  |  |
| Grade | I | 67 | 40 (59.70) | 27 (40.30) | 0.249 |
|  | II | 99 | 50 (50.51) | 49 (49.49) |  |
|  | III | 104 | 64 (61.54) | 40 (38.46) |  |
|  | n/a | 4 | 4 |  |  |
| Lymph node status | LNN | 95 | 60 (63.16) | 35 (36.84) | 0.393 |
|  | Node pos. | 127 | 73 (57.48) | 54 (42.52) |  |
|  | n/a | 52 | 25 | 27 |  |
| PgR | Positive | 184 | 100 (54.35) | 84 (45.65) | 0.112 |
|  | Negative | 90 | 58 (64.44) | 32 (35.56) |  |
|  | n/a | - |  |  |  |
| HER2 | Positive | 30 | 14 (46.67) | 16 (53.33) | 0.210 |
|  | Negative | 242 | 142 (58.68) | 100 (41.32) |  |
|  | n/a | 2 | 2 |  |  |
| DFS | Num. of events | 105 | 52 | 53 | 0.031^#^ |
|  | Mean (months) | 93 | 96 | 87 |  |
| OS | Num. of events | 96 | 47 | 49 | 0.027^#^ |
|  | Mean (months) | 96 | 100 | 91 |  |
| Abbreviations: LNN, Lymph node negative; PgR, Progesterone Receptor; HER2, human epidermal growth factor receptor-2 | | | | | |
| # Log-Rank test p-value. | | | | | |

| SD Table 7 – Comparison of the clinico-pathological characteristic of the cohorts | | | | |
| --- | --- | --- | --- | --- |
| **Parameter** | **Status** | **Luminal breast carcinoma cases** | | ***p*** |
|  |  | **Discovery Cohort (n=232)** | **Validation Cohort (n=274)** |  |
| Age | ≤ 50 | 94 (40.52) | 78 (28.47) | 0.004 |
|  | > 50 | 138 (59.48) | 196 (71.53) |  |
|  | n/a | - | - |  |
| Size | ≤ 2 cm | 123 (53.02) | 111 (40.51) | 0.005 |
|  | > 2 cm | 109 (46.98) | 163 (59.49) |  |
|  | n/a | - | - |  |
| Grade | I | 62 (26.84) | 67 (24.81) | 0.002 |
|  | II | 113 (48.92) | 99 (36.67) |  |
|  | III | 56 (24.24) | 104 (38.52) |  |
|  | n/a | 1 | 4 |  |
| Lymph node status | LNN | 108 (50.23) | 95 (42.79) | 0.119 |
|  | Node pos. | 107 (49.77) | 127 (57.21) |  |
|  | n/a | 17 | 52 |  |
| PgR | Positive | 94 (91.26) | 184 (67.15) | 1.000* |
|  | Negative | 9 (8.74) | 90 (32.85) |  |
|  | n/a | 129 | - |  |
| HER2 | Positive | 31 (13.36) | 30 (11.03) | 0.424 |
|  | Negative | 201 (86.64) | 242 (88.97) |  |
|  | n/a | - | 2 |  |
| Abbreviations: LNN, Lymph node negative; PgR, Progesterone Receptor; HER2, human epidermal growth factor receptor-2 | | | | |
| *Fisher exact test p-value. | | | | |

### SD Figure 1 – Survival probability according to copy number status on PPP2R2A and sensitivity analysis

No CNA (n=374)

CNA (n=208)

Probability of survival

Wilcoxon *p*= 0.047

Overall Survival (years)

As a sensitivity analysis, we performed the survival analysis only using as survival events the ones reported in patients who had reported recurrence/progression before the survival event. In this analysis, 21 survival events were registered (18 events in patients with CNA vs. 3 events in patients with no CNA) and 17 occurred in the first 70 months, thus supporting the assumption that disease-specific events occurred earlier. Again, the presence of CNA (either HetDel or HomDel) on PPP2R2A is significantly associated with survival (Wilcoxon p=0.047).

### SD Figure 2 – Survival probability according to Cyclin D1 (CCND1) expression status.

Cyclin D1 normal (n=110)

Probability of survival

Cyclin D1^high^ (n=109)

Log-Rank *p*= n.s.; HR=1.41 (0.67-2.95)

Log-Rank *p*= n.s. ; HR=1.30 (0.71-2.38)

Overall Survival (months)

Disease Free Survival (months)

Cyclin D1 normal (n=110)

Cyclin D1^high^ (n=109)

In this cohort of luminal breast cancer patients, despite slight display of worse prognosis in the Kaplan-Meier plots, no significant differences were found on the CCND1^high^ subgroup for both (a) OS and (b) DFS) (n=219).

### SD Figure 3 – Forrest plot with hazard-ratios for predictors of OS


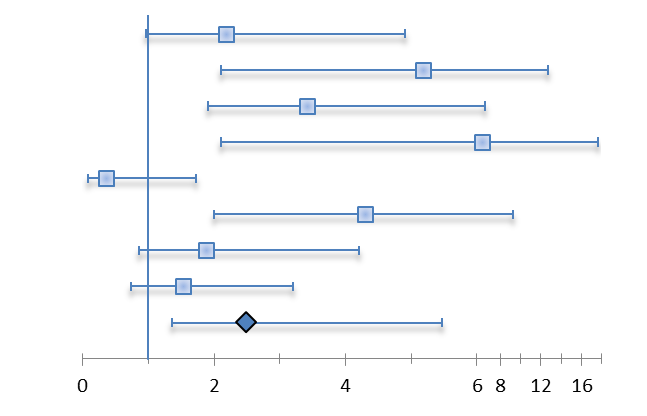


Size

Grade

LN status

Age

PgR

Her 2-OE

Ki-67

Cyclin D1^high^

PPP2R2A^-/low^ Cyclin D1^high^

Forrest plot depicting univariate hazard ratios (HR) with 95% confidence interval form a Cox-Proportional Hazards Model for the main clinico-pathological variables, Cyclin D1 expression and combined PPP2R2A/Cyclin D1 phenotype as predictors of overall survival (n=227).

## Survival Model of PPP2R2A^-/low^/Cyclin D1^high^ phenotype

In our survival analysis to assess the impact of the PPP2R2A^-/low^/Cyclin D1^high^ phenotype on survival, we observed that the PPP2R2A^-/low^/Cyclin D1^high^ phenotype was significantly associated with worse OS and DFS (Table 2).

Next we performed multiple imputation (20 imputations) for the 17 missing values in lymph node status. Lymph node status was imputed using tumor size, the PPP2R2A (B55α)/Cyclin D1 phenotype and HER2 status. Ki-67 index was not included because it was not a predictor of OS.

The association between the PPP2R2A^-/low^/Cyclin D1^high^ phenotype and OS/DFS was smaller when we adjusted for HER2 status, which can be potentially explained by association of these two variables with each other. To test this hypothesis, we ran a logistic regression model using the PPP2R2A^-/low^/Cyclin D1^high^ phenotype as a predictor of HER2 status. In this logistic regression we observe an HR of 3.37 (95%CI 1.24-9.13) with a p-value 0.017, thus confirming occurrence of co-linearity between these two variables and explaining the impact on survival. Nonetheless, the phenotype PPP2R2A^-/low^/Cyclin D1^high^ increases upon our prognostic ability.

# Supplementary Data References

1. Reis-Filho JS, Milanezi F, Carvalho S, Simpson PT, Steele D, Savage K, Lambros MB, Pereira EM, Nesland JM, Lakhani SR *et al*: **Metaplastic breast carcinomas exhibit EGFR, but not HER2, gene amplification and overexpression: immunohistochemical and chromogenic in situ hybridization analysis**. *Breast cancer research : BCR* 2005, **7**(6):R1028-1035.

2. Martins D, Beca FF, Sousa B, Baltazar F, Paredes J, Schmitt F: **Loss of caveolin-1 and gain of MCT4 expression in the tumor stroma: key events in the progression from an in situ to an invasive breast carcinoma**. *Cell cycle* 2013, **12**(16):2684-2690.

3. Witkiewicz AK, Whitaker-Menezes D, Dasgupta A, Philp NJ, Lin Z, Gandara R, Sneddon S, Martinez-Outschoorn UE, Sotgia F, Lisanti MP: **Using the "reverse Warburg effect" to identify high-risk breast cancer patients: stromal MCT4 predicts poor clinical outcome in triple-negative breast cancers**. *Cell cycle* 2012, **11**(6):1108-1117.

4. Mazumdar M, Glassman JR: **Categorizing a prognostic variable: review of methods, code for easy implementation and applications to decision-making about cancer treatments**. *Statistics in medicine* 2000, **19**(1):113-132.
